# Land snails can trap trematode cercariae in their shell: Encapsulation as a general response against parasites?[Fn FN1]

**DOI:** 10.1051/parasite/2023001

**Published:** 2023-01-19

**Authors:** Claudia Gérard, Youna De Tombeur, Maxime Dahirel, Armelle Ansart

**Affiliations:** 1 Université de Rennes, UR1, CNRS, ECOBIO (Ecosystèmes, Biodiversité, évolution), UMR 6553 35000 Rennes France; 2 Department of Biology, Ghent University 9000 Ghent Belgium

**Keywords:** Helicids, Cercariae, Shell-encapsulation, Defence response

## Abstract

Terrestrial gastropods are hosts of a wide variety of metazoan parasites and can respond to parasite exposure in various ways. One of these defence mechanisms, the ability to trap parasites in the host shell, was previously thought to apply only against nematodes. During a field survey along an urbanisation gradient, we found that the shell of *Cornu aspersum* and *Cepaea nemoralis* can contain encapsulated trematode cercariae, with prevalences of 7% and 1%, respectively over the entire sample, and up to 47% at the local population level. To our knowledge, this is the first case study unambiguously showing that land snails can trap non-nematode parasites in their shell at non-negligible prevalences. Shell-encapsulation could be a more general defence mechanism than previously described, and more studies are needed to understand its importance and variability.

## Abstract (fr)

Les gastéropodes terrestres sont les hôtes d’une grande variété de métazoaires parasites, et peuvent répondre de plusieurs façons à l’exposition parasitaire. L’un de ces mécanismes de défense, la capacité à piéger le parasite dans la coquille, semblait ne concerner que les nématodes. Lors d’un suivi de terrain le long d’un gradient d’urbanisation, nous avons observé que la coquille de *Cornu aspersum* et de *Cepaea nemoralis* pouvait contenir des cercaires de trématodes encapsulées, à des prévalences respectives de 7 % et 1 % sur l’ensemble de l’échantillon, et pouvant atteindre 47 % au niveau d’une population locale. À notre connaissance, ceci est la première étude montrant sans ambiguïté que les escargots terrestres peuvent piéger de façon significative des parasites autres que les nématodes dans leur coquille. L’encapsulation des parasites dans la coquille pourrait donc être un mécanisme de défense plus général que précédemment décrit, plus d’études étant nécessaires pour comprendre son importance et sa variabilité.

## Introduction

Terrestrial molluscs (land snails and slugs) are hosts of a large diversity of metazoan parasites, both on the phylogenetic (nematodes, trematodes, mites, and insects) and life-history axes (obligate vs. facultative parasites, parasitoids, with complex life cycles, with a free-living phase, and sexually transmitted parasites) [3, 15, 20, 24]. Their defence mechanisms and immune responses against these parasites are also diverse and may involve a combination of behavioural and physiological responses (e.g. [6, 14, 25]). Physiological responses include, for instance, phagocytosis or encapsulation by haemocytes (for review: [14]), likely mediated by the melanin/phenoloxidase pathway [6]. Additionally, it has been shown that land snails exposed to parasitic nematodes can defend themselves by trapping them in their shell [7, 13, 22, 23]. In this case, cells on the inner layer of the snail shell adhere to the cuticle of nematodes, engulf their body and fuse them to the inside of the shell [13, 22]. This ability is remarkably conserved across the terrestrial gastropod phylogeny, even being present in some slugs with a vestigial internal shell [22]. Based on extensive surveys failing to find other parasites, it was thought that shell-encapsulation is a specific defence mechanism against nematodes [22]. We recently found two *Cepaea nemoralis* individuals that had trapped parasitic mites (*Riccardoella* sp.) in their shell [9]. However, it remains uncertain whether this is a rare event or if this result shows that shell-encapsulation is not nematode-specific. In addition, mites may not even have been alive at trapping time, and that observation may reflect a general response to inert foreign bodies rather than active defence. Alongside nematodes, trematodes are frequent parasites of gastropods, including terrestrial ones, and use them as first and/or second intermediate hosts [1, 20, 24]. In a typical life cycle, trematode adults, living inside definitive vertebrate hosts, release eggs into the environment [21]. The miracidia hatch from these eggs and infect a molluscan first intermediate host [21]. Following asexual amplification of larval stages inside sporocysts or rediae, cercariae leave the mollusc and penetrate a second intermediate host to encyst as metacercariae [21]. The cycle is completed when this second intermediate host is ingested by the definitive host [21]. Snails can mobilise haemolymph immune defence mechanisms, such as encapsulation by haemocytes, against larval trematodes, but the efficiency varies depending on species (incompatible vs. compatible), probably in relation to similarity between snail haemocytes and the surface carbohydrates of parasites [1]. Given this, trematodes, which may be present in the space between the shell and soft body at the cercarial stage when leaving the first intermediate host or reaching the second one, are a good target to determine whether shell-encapsulation is taxon-specific.

The helicids *Cornu aspersum*, *Cepaea hortensis* and *C. nemoralis* are some of the most studied snail species in Europe, and can be infected by various metazoan parasites such as nematodes, trematodes and mites (e.g. [3, 9, 15, 27]). In this study, we used data from a range of local populations of these three species to determine whether they were able to use their shell as a defence system to encase and kill metazoan parasites other than nematodes, and especially trematodes.

## Materials and methods

In the course of a previously planned urban ecology study (Dahirel et al., unpublished), we visited 20 sites along an urbanisation gradient in Rennes (Brittany, France, ≈48°7^′^ N 1°40^′^ W) in April 2022. We sampled live adult snails, focusing on the family Helicidae. Adult helicids are recognisable by the presence of a reflected “lip” around the shell opening. We collected a total of 303 *C. aspersum* snails (at 17/20 sites), 74 *C. nemoralis* (at 4/20 sites) and 15 *C. hortensis* (at 1/20 sites) overall, with between 10 and 20 individuals per snail species per site where the species was found. After the experiments planned in the aforementioned study, all the snails were frozen (−20 °C) and then thawed to search for metazoan parasites in the shell and in various locations (space between body and shell, genital and digestive systems, kidney, lung, heart) under stereoscopic microscope with backlighting as in previous studies [9, 15]. Larval stages of the trematode genus *Brachylaima* Dujardin, 1843 were morphologically identified according to Gracenea and González-Moreno [17] and Segade et al. [24]. We described parasitism by prevalence (P%) (number of hosts infected with a particular parasite species/number of examined hosts) [5].

## Results

**Figure 1:**
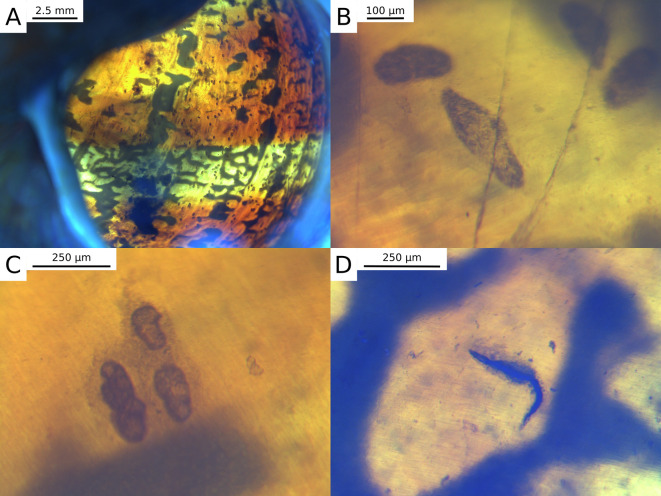
Backlit views of metazoan parasites trapped in the shell of *Cornu aspersum*: trematode cercariae (A, B, C), and nematode (D). Small cracks of the inner shell layer (B) can be seen above the cercariae and were considered indicative of damage on the shell after it covered cercariae. Note the accumulation of dark adhering cells around or above the parasites for both cercariae (C) and nematodes (D). High-resolution versions of these images are archived in Zenodo (https://doi.org/10.5281/zenodo.7376465).

Overall, 43.2% (131/303) of *C. aspersum*, 44.6% (33/74) of *C. nemoralis* and 6.7% (1/15) of *C. hortensis* harboured live metazoan parasites in their body, mainly trematodes *Brachylaima* sp. (see Table 1 for details). *Brachylaima* sp. individuals were usually metacercariae, but other larval stages (sporocysts and cercariae) were also found live in snails. A greater proportion of snails, 59.4% (180/303), 83.8% (62/74) and 93.3% (14/15), respectively for the three host species, had dead parasites trapped in their shell (Table 1, Fig. 1). While these trapped parasites were mostly nematodes (Fig. 1D), we also recorded trematode cercariae in *C. aspersum* (21/303) and *C. nemoralis* (1/74; Table 1). Trematodes appeared to be clearly trapped in the shell (Fig. 1B) and surrounded by adhering shell cells as observed in encased nematodes (compare Figs. 1C and 1D). Among these 22 snails, the median number of trapped cercariae per individual was five, but the shell of three individuals, all *C. aspersum*, contained ≥100 cercariae each (Fig. 1A). Shell-trapped cercariae probably belonged to *Brachylaima* sp. based on morphological resemblances with live cercariae (Fig. 1B). In addition, *Brachylaima* was the only trematode genus found parasitizing snails in our samples, and the majority of snails with encased cercariae (13/22) also harboured live *Brachylaima* larvae. In *C. aspersum*, there was among-population variation in the prevalence of trematode encapsulation (Bernoulli GLM with a population of origin effect, $\chi_{16}^{2}=83.2$, *p* = 4.4 × 10^−11^) with only 7/17 populations presenting shell-encapsulated trematodes (population-level prevalences from 5.0% to 47.1%).

**Table 1:** Prevalence of metazoan parasites (live and shell-trapped) in three helicid species: *Cornu aspersum*, *Cepaea nemoralis* and *Cepaea hortensis*. Estimates are based on pooling all sites together.

	*Cornu aspersum*	*Cepaea nemoralis*	*Cepaea hortensis*
Number of snails sampled across all 20 sites	303	74	15
Number of sites with snail species present	17	4	1
− in which shell-trapped trematode cercariae were found	7	1	0
*Prevalence, all sites pooled, of shell-trapped parasites*:			
− all metazoan parasites (trematodes and nematodes)	59.4%	83.8%	93.3%
− trematode cercariae	6.9%	1.4%	0.0%
− nematodes	56.4%	83.8%	93.3%
*Prevalence, all sites pooled, of live parasites*:			
− all metazoan parasites	43.2%	44.6%	6.7%
− trematode sporocysts/cercariae (in digestive gland)	6.6%	1.4%	0.0%
− trematode metacercariae (in kidney)	31.7%	33.8%	6.7%
− nematodes (in genitalia, lung, space or body-shell space)	13.9%	0.0%	0.0%
− mites (in lung)	0.0%	10.8%	0.0%

## Discussion

Our study demonstrates that trematode cercariae can be trapped in the shell of terrestrial gastropods, specifically the helicids *C. aspersum* and *C. nemoralis*. Combined with more anecdotal accounts showing that mites may also be shell-trapped [9]; this suggests that this host defence response to parasite exposure may not be limited to nematodes and appears to be more general than previously believed [22]. In the case of mites, shell-encapsulation probably occurred after death (or molting) based on their greater motility and size, and may not be a direct defence reaction to live parasites as for nematodes. In the case of the trematodes observed here, the fact that they elicited similar cellular aggregations to those seen in response to nematodes (Fig. 1, [22]) and the number of observations suggest they were indeed encased alive as a defence response. Nonetheless, even though non-nematode parasites are only trapped when dead, our observations still have interesting implications for our ability to track parasitism patterns from empty shells, including from historical collections. Morphological aspects of shell-trapped cercariae suggest that they belong to *Brachylaima*, a trematode genus we recorded alive at different larval stages (sporocysts, cercariae, and metacercariae) in the present study, and which was already known to infect various helicid snails (e.g. [9, 15, 17, 27]). Before reaching a definitive vertebrate host, the life cycle of *Brachylaima* includes two successive land snails (first and second intermediate hosts), which can be of the same species but without possible autoinfection of the same host individual [24]. Cercariae produced by sporocysts in the digestive gland emerge from the first intermediate snail host depending on water availability, and crawl actively on the humid substrate until they contact the second one; then they enter the host kidney via the ureter and develop into non-encysted fully mature metacercariae [24]. Despite probable limited displacements of terrestrial crawling cercariae, massive metacercarial infections can occur (e.g., in this study, up to 150 metacercariae in the same host individual). The ability to encapsulate cercariae in their shell would allow helicids to prevent metacercarial infection from reaching critical numbers in the kidney. Importantly, extensive necrotic changes occur in the renal epithelium in the presence of numerous actively feeding metacercariae of *Brachylaima* [24]. Alternatively, cercariae might be shell-trapped by the first intermediate host after their release from its digestive gland and before ever reaching a new host. Further investigations are needed to pinpoint when cercariae are shell-trapped in their life cycle, as the ecological, epidemiological and evolutionary implications are likely to differ widely depending on whether the first or second host is most often involved.

As parasite encapsulation in the shell is evolutionarily conserved across land gastropods (demonstrated for nematodes) [22] and appears to be a relatively general response to metazoan parasites rather than a specialised response to specific taxa, our finding raises several other key questions for our understanding of mollusc-parasite interactions. Firstly, there seems to be substantial variation in shell-encapsulation rates among populations of the same snail species and between parasite taxa ([7, 9, 13, 22, 23], this study). So far, studies of shell-encapsulation in wild land snail populations have been relatively limited in the number of populations studied. More taxonomically and geographically widespread surveys would help us understand the extent of this variability, its correlation with variation in parasite distribution, and whether shell-encapsulation propensity can be predicted from host and/or parasite traits. Second, comparing this defence mechanism to similar systems in more distantly related mollusc taxa may help us understand its evolution. Similarly, the implication of different trematode families in the formation of pearls and other shell material concretions has been described in numerous fossil and modern bivalves (e.g. [4, 8, 10, 16, 19]) and cephalopods [11, 12, 18]. Moreover, genes involved in bivalve shell growth and biomineralisation are upregulated after immune challenge [26]. While to our knowledge, trematodes had not been implicated in shell-encapsulation mechanisms in gastropods, aquatic or terrestrial, until the present study, a pearl-like formation had been observed in the land snail *Canistrum ovoideum* (family Bradybaenidae) that could be of a pathological origin [2]. Investigating the shared physiological underpinnings of these various responses, if any, could lead us to consider the ability to engulf and inhibit metazoan parasites using shell material as a general defence mechanism of mollusca.
